# Association of measured physical performance and demographic and health characteristics with self-reported physical function: implications for the interpretation of self-reported limitations

**DOI:** 10.1186/1477-7525-8-84

**Published:** 2010-08-13

**Authors:** Grant H Louie, Michael M Ward

**Affiliations:** 1 Intramural Research Program, National Institute of Arthritis and Musculoskeletal and Skin Diseases, National Institutes of Health, Bethesda, Maryland, USA

## Abstract

**Background:**

Self-reported limitations in physical function often have only weak associations with measured performance on physical tests, suggesting that factors other than performance commonly influence self-reports. We tested if personal or health characteristics influenced self-reported limitations in three tasks, controlling for measured performance on these tasks.

**Methods:**

We used cross-sectional data on adults aged ≥ 60 years (*N *= 5396) from the Third National Health and Nutrition Examination Survey to examine the association between the repeated chair rise test and self-reported difficulty rising from a chair. We then tested if personal characteristics, health indicators, body composition, and performance on unrelated tasks were associated with self-reported limitations in this task. We used the same approach to examine associations between personal and health characteristics and self-reported difficulty walking between rooms, controlling for timed 8-foot walk, and self-reported difficulty getting out of bed, controlling for repeated chair rise test results.

**Results:**

In multivariate analyses, participants who performed worse on the repeated chair rise test were more likely to report difficulty with chair rise. However, older age, lower education level, lower serum albumin, comorbidities, knee pain, and being underweight were also significantly associated with self-reported limitations with chair rise. Results were similar for difficulty walking between rooms and getting out of bed.

**Conclusions:**

Self-reports of limitations in physical function are influenced by personal and health characteristics that reflect frailty, and should not be interpreted solely as measured difficulty performing the task.

## Background

Physical functioning is a key component of health-related quality of life (HRQL) [1]. Attention to limitations in physical functioning is increasing in clinical practice because these limitations are important to patients, diminish HRQL, and predict future health outcomes and the need for care [1-10].

A gold standard method to measure physical functioning does not exist. Self-report questionnaires have been adopted as easily administered instruments that can capture limitations in a wide spectrum of tasks [11,12]. However, self-report is subjective and may be influenced by mood, misjudgment of usual ability, or misinterpretation by the respondent. Despite these potential limitations, self-report questionnaires of physical functioning have face and construct validity [2]. An approach commonly used to test the construct validity of self-reported measures of functioning is to compare responses on these measures with directly-observed or measured performance on similar tasks. For example, self-reported difficulty in rising from a chair is tested for correlations with measured ability to rise from a chair on a timed test.

Although self-reported functioning and performance on objective physical tests are correlated, these associations are generally weak [13-17]. These studies often examined associations between multi-item questionnaires and physical performance test batteries, which averaged measures of performance over several domains of functioning [14,15,18-20]. One explanation for the weak correlations in these studies may be the problem of compensability: multi-item or summary measures do not identify which functions are most limited, the mean result might compensate for isolated limitations, and good performance on some measures might confound the association between other performance measures and their corresponding self-reported functions. To use physical performance tests to assess the construct validity of self-reported measures, it would be more appropriate to compare highly specific pairings of physical performance tests and self-reported physical function; that is, how self-reported limitations compare to measured performance on a corresponding test of the same task.

An alternative explanation for the modest association between self-report and physical performance tests may be that factors other than performance affect self-reports of limitations. Personal and health characteristics may influence how different patients appraise the limitations they have.

Despite extensive literature on the use of self-reports to measure physical functioning, few studies to date have examined whether factors other than measured performance on the same task influence self-reports. Our primary objective was to determine if self-reported limitations in physical functions were associated with personal and health characteristics, after accounting for measured performance on the same task. To address the problem of compensability, we analyzed three self-report tasks (rising from a chair, getting in or out of bed, and walking between rooms) for which there were corresponding physical performance tests (timed repeated chair rise and 8-foot walk). This design provided a unique method with which to assess influences on, and the meaning of, self-reported limitations.

## Methods

### Data source and study sample

We analyzed data from the Third National Health and Nutrition Examination Survey (NHANES III), a national population-based sample of non-institutionalized individuals in the United States [21]. In this cross-sectional study, we included participants aged ≥ 60 years because only these persons were eligible for an assessment of physical function. Among this subset, we excluded from the study individuals (n = 441) who lacked assessment of physical function at the mobile examination center (92.3%) or their home (7.7%) by one of the following three physical performance tests: repeated chair rise, 8-foot walk, or lock and key test. Our final study sample included 5396 persons. Participants completed the Household Questionnaire, which included questions on physical functioning, before they had the physical examination and performance testing.

### Analytic framework

To maximize the specificity of the association between physical performance tests and self-reported physical function, we studied performance tests in relation to their corresponding self-reported functions: 1) repeated chair rise test and its relationship with self-reported difficulty rising from a chair; 2) repeated chair rise test and its relationship with self-reported difficulty getting in or out of bed; and 3) 8-foot walk test and its relationship with self-reported difficulty walking between rooms on the same level. Limitations on eight additional physical functions were asked, but were not included in the analysis because they did not have a corresponding physical performance test administered in NHANES III.

### Dependent variables

Ability to perform the three self-reported physical functions of interest was assessed by the following: "Please tell me if you have no difficulty, some difficulty, much difficulty or are unable to do these activities at all when you are by yourself and without the use of aids." 1) "Standing up from an armless straight chair?", 2) "Getting in or out of bed?", and 3) "Walking from one room to another on the same level?".

### Independent variables

Data on six physical performance tests were collected in NHANES III by trained assessors: repeated chair rise test, 8-foot walk, lock and key test, shoulder range of motion, active hip and knee flexion, and timed tandem stand test [16,22-25]. The repeated chair rise test, an assessment of lower extremity motor function and postural control, was a timed test of five consecutive rises from an armless straight chair. The 8-foot walk test, an evaluation of gait, was a timed test of usual speed to walk 8 feet. Time to complete the test (in seconds) was represented as gait speed (in meters per second) by first converting feet to meters and then dividing by the time in seconds needed to complete the test. We categorized performance on both tests into quartiles, with the best-performing quartile as the reference group.

The lock and key test, a test of eye-hand coordination and fine motor skills, was a timed test of unlocking a lock with a key. Internal and external rotations of both shoulders were scored as full, partial, or unable to perform. Hip and knee flexion were scored similarly. For analysis, results on the lock and key test were categorized into quartiles, and range of motion of the shoulders and flexion of the hips and knees were dichotomized as either full or not, with full as the reference group. We did not include the timed tandem stand test because almost all participants attained the maximum allotted time. Reliability of these physical performance tests has been reported to be good [16,22-25].

We included covariates available in the data set and known to be associated with physical function. Demographic characteristics included age, gender, race-ethnicity, and education level. We categorized age into five groups (60-64, 65-69, 70-74, 75-79, and 80 years and older) to allow for non-linear relationships, and categorized education level, recorded as highest grade attained, into three groups (0-8, 9-12, and 13-17 years).

The health indicators were current cigarette smoking, hemoglobin level, serum albumin concentration, knee pain, and comorbidities. We included current cigarette smoking, hemoglobin, and serum albumin because they are indicators of general health [26-29]. Hemoglobin and serum albumin were used as continuous variables in the regression models, with associated odds ratios representing change per 1 gram per deciliter. Knee pain was included because the functions we studied involved the lower extremities, and pain may affect physical function. Knee pain, recorded as tenderness on palpation or pain with passive motion during the physical examination, was coded as absent, present in one knee, or present in both knees. We included comorbidities that may impact physical function: arthritis, stroke, diabetes mellitus, chronic bronchitis, emphysema, asthma, myocardial infarction, congestive heart failure, and cancer (excluding skin cancer). These were collected by self-report.

Body composition was assessed by body mass index (BMI) and skeletal muscle mass, which are prognostic indicators of physical function. BMI, measured as weight in kilograms/height in meters squared, was grouped using World Health Organization categories of underweight (< 18.5 kg/m^2^), normal weight (18.5 - 24.9 kg/m^2^), overweight (25.0 - 29.9 kg/m^2^), and obesity (≥ 30.0 kg/m^2^) because of its non-linear relationship with physical function [30,31]. Skeletal muscle mass was determined from a prediction equation based on bioelectrical impedance analysis resistance, age, gender, and height. Following Janssen, we expressed skeletal muscle mass as skeletal muscle index (SMI) to account for differences in non-skeletal muscle mass, where SMI = (skeletal muscle mass/body mass) × 100 [32].

### Statistical analysis

Analyses were performed using methods that accounted for the multistage, clustered sampling of NHANES III. We used ordinal logistic regression models to examine the association between specific physical performance tests and self-reported limitations. In unadjusted models, the degree of self-reported functional limitation was the dependent variable and the corresponding physical performance test was the independent variable. In adjusted models, we included age, gender, race-ethnicity, education level, arthritis, stroke, diabetes mellitus, chronic bronchitis, emphysema, asthma, myocardial infarction, congestive heart failure, cancer, smoking, hemoglobin level, serum albumin concentration, knee pain, BMI, and SMI. To determine if other physical performance tests were associated with any of the three self-reported functional limitations, we then added the lock and key test, shoulder range of motion, hip and knee flexion, and either chair rise test or 8-foot walk test as independent variables to each model.

To assess the validity of the proportional odds assumption in the ordinal logistic regression models, we examined qualitatively the similarity of odds ratios for contrasts between each level of the dependent variable [33]. We represented the associations with a single odds ratio, since odds ratios for different contrasts were found to be similar.

Data were missing for education level in 0.7% of cases, height in 0.2%, weight in 0.3%, hemoglobin in 5.9%, serum albumin in 7.8%, bioelectrical impedance analysis resistance in 17.3%, chair rise test in 10.3%, 8-foot walk test in 7.1%, lock and key test in 4.5%, shoulder rotation in 0.3%, and hip and knee flexion in 6.3%. Data were missing due to different reasons. During evaluation of physical functioning, participants who made no attempt to perform a specific maneuver because of severe physical limitations were coded as "blank". We assigned these participants to the worst performing quartile. Participants who attempted the task but failed to complete it were also assigned to the worst performing quartile. On the other hand, participants who made no attempt to perform a specific maneuver for reasons unrelated to physical limitations (e.g. time constraints) were coded as "blank but applicable". After extensive review by survey analysts, data believed to be extreme or illogical and viewed as virtually impossible were also coded as "blank but applicable". We treated data coded as blank but applicable as missing at random. We used the multiple imputation method with the Markov Chain Monte Carlo algorithm to impute missing values [34]. This allowed us to retain all participants in the analyses, and provides estimates that are less biased than those of a complete-case analysis [35]. Analyses were performed using SAS version 9.2 (SAS Institute Inc, Cary, NC).

## Results

### Participant characteristics

Participants had a mean (± standard error of the mean) age of 70.7 ± 0.2 years (**Table **1). Arthritis was the most common comorbid condition (44.7%), while 12.6% reported having diabetes mellitus, and 11.4% reported having had a myocardial infarction. At least some difficulty rising from an armless straight chair was reported by 20.5%; 14.9% reported at least some difficulty getting in or out of bed, and 8.2% reported at least some difficulty walking between rooms on the same level.

**Table 1:** Characteristics of the Participants

	Percent
Age, y	
60-64	25.2
65-69	24.7
70-74	20.5
75-79	14.4
≥ 80	15.2
Women	57.3
Race-ethnicity*	
Non-Hispanic White	84.7
Non-Hispanic Black	8.3
Mexican-American	2.3
Other	14.7
Education level^†^, y	
≥ 13	27.4
9-12	47.9
0-8	24.7
Arthritis	44.7
Stroke	6.8
Diabetes mellitus	12.6
Chronic bronchitis	9.3
Emphysema	5.9
Asthma	7.1
Myocardial infarction	11.4
Congestive heart failure	7.2
Cancer (excluding skin cancer)	9.4
Current cigarette smoking	15.3
Hemoglobin^‡^, g/dL	13.93 ± 0.03
Serum albumin^‡^, g/dL	4.04 ± 0.02
Body mass index, kg/m^2^	
< 18.5	2.6
18.5-24.9	35.6
25-29.9	38.7
≥ 30	23.1
Skeletal muscle index^‡^, %	31.11 ± 0.11

*Race-ethnicity self-reported.

^†^Highest grade or year of regular school completed.

^‡^Plus-minus values are mean ± standard error of the mean.

Compared to participants included in the study, those who were excluded due to lack of assessment of physical function by physical performance tests had similar demographic characteristics. They had a mean age of 71.6 ± 0.6 years (vs. 70.7 ± 0.2 years), were mostly women (58.9% vs. 57.3%), and non-Hispanic white (81.0% vs. 84.7%). They generally had more comorbidities, with a higher proportion reporting ever been diagnosed with arthritis (46.7% vs. 44.7%), stroke (11.7% vs. 6.8%), diabetes mellitus (14.5% vs. 12.6%), and chronic bronchitis (12.6% vs. 9.3%).

### Association of physical performance tests with self-reported functional limitations

In the first set of analyses that tested the association of the repeated chair rise test and self-reported limitations rising from a chair, worse performance on the chair rise test was significantly associated with the odds of reporting worse limitations (**Table **2). In adjusted models, age was a significant correlate of functional limitation, independent of performance on the repeated chair rise test, with progressively higher adjusted odds ratios beginning with 70-74 year-olds. Lower education level, arthritis, stroke, congestive heart failure, and cancer were associated with a higher odds of worse self-reported limitation, while a higher level of serum albumin was associated with a lower odds of a worse self-reported limitation. Participants with bilateral knee pain and those who were underweight or obese were more likely to report worse limitations. Gender, current smoking, hemoglobin level, and SMI were not associated with self-reported limitation in rising from a chair in this model.

**Table 2:** Association of Self-Reported Ability to Rise from Armless Straight Chair with Chair Rise Test

	*Unadjusted*			*Adjusted*		
	**OR**	**95% CI**	**P**	**OR**	**95% CI**	**P**
		
Chair rise*, sec						
Q1 (2.0-11.1)	1.00			1.00		
Q2 (11.2-13.6)	1.55	1.13-2.14	0.008	1.31	0.95-1.82	0.10
Q3 (13.7-17.5)	2.67	1.98--3.60	< 0.0001	1.87	1.37-2.56	0.0002
Q4 (17.6-100)	8.83	6.46-12.08	< 0.0001	4.43	3.17-6.20	< 0.0001
						
Age, y						
60-64				1.00		
65-69				1.19	0.80-1.78	0.39
70-74				1.62	1.15-2.30	0.006
75-79				1.96	1.39-2.75	0.0001
≥ 80				3.51	2.52-4.90	< 0.0001
						
Men				1.00		
Women				0.87	0.59-1.27	0.46
						
Race-ethnicity						
Non-Hispanic White				1.00		
Non-Hispanic Black				0.70	0.53-0.92	0.01
Mexican-American				1.16	0.83-1.62	0.39
Other				1.16	0.68-1.98	0.59
						
Education, y						
≥ 13				1.00		
9-12				1.01	0.75-1.34	0.96
0-8				1.37	1.03-1.81	0.03
						
Arthritis						
No				1.00		
Yes				2.43	1.99-2.93	< 0.0001
						
Stroke						
No				1.00		
Yes				2.58	1.84-3.61	< 0.0001
						
Diabetes mellitus						
No				1.00		
Yes				1.13	0.87-1.47	0.37
						
Chronic bronchitis						
No				1.00		
Yes				1.07	0.74-1.54	0.73
						
Emphysema						
No				1.00		
Yes				1.07	0.66-1.72	0.79
						
Asthma						
No				1.00		
Yes				0.83	0.51-1.33	0.44
						
Myocardial infarction						
No				1.00		
Yes				1.27	0.96-1.68	0.10
						
Congestive heart failure						
No				1.00		
Yes				1.68	1.21-2.32	0.002
						
Cancer (excluding skin cancer)						
No				1.00		
Yes				1.36	1.01-1.84	0.04
						
Current smoking						
No				1.00		
Yes				1.22	0.90-1.65	0.20
						
Hemoglobin, g/dL				0.94	0.87-1.02	0.11
						
Serum albumin, g/dL				0.45	0.32-0.63	< 0.0001
						
Painful knees, no.						
0				1.00		
1				1.48	0.99-2.21	0.06
2				1.82	1.29-2.56	0.0006
						
BMI^†^, kg/m^2^						
< 18.5				2.45	1.60-3.75	< 0.0001
18.5-24.9				1.00		
25-29.9				1.18	0.87-1.60	0.27
≥ 30				1.38	1.01-1.88	0.04
						
SMI^‡^, %				0.98	0.95-1.01	0.18

*Q1, 2, 3, 4 represent 1^st ^through 4^th^quartiles, from best performance (Q1) to worst performance (Q4).

^†^BMI = body mass index.

^‡ ^SMI = skeletal muscle index.

Results of models predicting self-reported ability to get in or out of bed were similar (**Table **3). Participants in the 3^rd ^and 4^th ^quartiles on the chair rise test were more likely to report worse limitations than those in the best-performing quartile. In the adjusted model, older age, lower education level, arthritis, stroke, congestive heart failure, cancer, lower serum albumin level, bilateral knee pain, and lower BMI were also significantly associated with an increased odds of worse self-reported functioning, independent of measured performance on the chair rise test.

**Table 3:** Association of Self-Reported Ability to Get In or Out of Bed and Chair Rise Test

	*Unadjusted*			*Adjusted*		
	**OR**	**95% CI**	**P**	**OR**	**95% CI**	**P**
		
Chair rise*, sec						
Q1 (2.0-11.1)	1.00			1.00		
Q2 (11.2-13.6)	1.12	0.74-1.72	0.58	0.98	0.64-1.49	0.91
Q3 (13.7-17.5)	2.27	1.45-3.57	0.001	1.70	1.08-2.69	0.02
Q4 (17.6-100)	6.92	4.88-9.81	< 0.0001	3.84	2.74-5.39	< 0.0001
						
Age, y						
60-64				1.00		
65-69				0.93	0.58-1.47	0.75
70-74				1.02	0.71-1.45	0.92
75-79				1.05	0.71-1.57	0.80
≥ 80				1.65	1.13-2.41	0.009
						
Men				1.00		
Women				0.74	0.48-1.12	0.15
						
Race-ethnicity						
Non-Hispanic White				1.00		
Non-Hispanic Black				0.70	0.52-0.96	0.03
Mexican-American				1.20	0.83-1.73	0.32
Other				1.08	0.67-1.74	0.76
						
Education, y						
≥ 13				1.00		
9-12				1.03	0.72-1.47	0.89
0-8				1.57	1.08-2.27	0.02
						
Arthritis						
No				1.00		
Yes				2.75	2.19-3.43	< 0.0001
						
Stroke						
No				1.00		
Yes				2.41	1.65-3.54	< 0.0001
						
Diabetes mellitus						
No				1.00		
Yes				1.23	0.97-1.56	0.09
						
Chronic bronchitis						
No				1.00		
Yes				1.26	0.92-1.72	0.15
						
Emphysema						
No				1.00		
Yes				1.40	0.85-2.33	0.19
						
Asthma						
No				1.00		
Yes				1.02	0.71-1.46	0.92
						
Myocardial infarction						
No				1.00		
Yes				1.14	0.80-1.62	0.47
						
Congestive heart failure						
No				1.00		
Yes				2.11	1.56-2.85	< 0.0001
						
Cancer (excluding skin cancer)						
No				1.00		
Yes				1.48	1.10-2.00	0.01
						
Current smoking						
No				1.00		
Yes				1.33	0.93-1.91	0.12
						
Hemoglobin, g/dL				1.02	0.92-1.12	0.74
						
Serum albumin, g/dL				0.54	0.37-0.78	0.001
						
Painful knees, no.						
0				1.00		
1				1.36	0.96-1.92	0.09
2				2.18	1.45-3.28	0.0002
						
BMI^†^, kg/m^2^						
< 18.5				2.51	1.36-4.62	0.003
18.5-24.9				1.00		
25-29.9				0.93	0.72-1.19	0.57
≥ 30				1.07	0.78-1.46	0.69
						
SMI^‡^, %				0.99	0.95-1.02	0.46

*Q1, 2, 3, 4 represent 1^st ^through 4^th ^quartiles, from best performance (Q1) to worst performance (Q4).

^†^BMI = body mass index.

^‡^SMI = skeletal muscle index.

In the third set of models examining self-reported ability to walk between rooms, the 8-foot walk test was significantly associated with the odds of a worse level of self-reported limitation (**Table **4). In the adjusted model, older age, arthritis, stroke, chronic bronchitis, congestive heart failure, cancer, and lower serum albumin level were significantly associated with self-reported limitation in walking between rooms, independent of measured performance on the 8-foot walk test.

**Table 4:** Association of Self-Reported Ability to Walk from One Room to Another on Same Level and 8-ft Walk Test

	*Unadjusted*			*Adjusted*		
	**OR**	**95% CI**	**P**	**OR**	**95% CI**	**P**
		
8-ft walk*, m/sec						
Q1 (≥ 0.91)	1.00			1.00		
Q2 (0.71-0.90)	1.29	0.62-2.68	0.49	1.25	0.60-2.62	0.55
Q3 (0.54-0.70)	3.23	1.62-6.43	0.0009	2.54	1.16-5.59	0.02
Q4 (≤ 0.53)	21.91	11.64-40.24	< 0.0001	14.20	6.67-30.24	< 0.0001
						
Age, y						
60-64				1.00		
65-69				1.67	0.85-3.39	0.14
70-74				1.87	1.03-3.40	0.04
75-79				1.50	0.84-2.68	0.17
≥ 80				2.10	1.13-3.92	0.02
						
Men				1.00		
Women				0.91	0.52-1.58	0.73
						
Race-ethnicity						
Non-Hispanic White				1.00		
Non-Hispanic Black				1.04	0.73-1.49	0.81
Mexican-American				1.16	0.70-1.92	0.56
Other				0.81	0.45-1.46	0.49
						
Education, y						
≥ 13				1.00		
9-12				0.75	0.48-1.17	0.20
0-8				0.94	0.61-1.43	0.77
						
Arthritis						
No				1.00		
Yes				2.86	2.17-3.77	< 0.0001
						
Stroke						
No				1.00		
Yes				2.26	1.56-3.26	< 0.0001
						
Diabetes mellitus						
No				1.00		
Yes				0.98	0.70-1.37	0.89
						
Chronic bronchitis						
No				1.00		
Yes				1.64	1.03-2.62	0.04
						
Emphysema						
No				1.00		
Yes				1.10	0.54-2.24	0.79
						
Asthma						
No				1.00		
Yes				0.88	0.54-1.42	0.60
						
Myocardial infarction						
No				1.00		
Yes				0.67	0.43-1.04	0.08
						
Congestive heart failure						
No				1.00		
Yes				2.57	1.52-4.33	0.0004
						
Cancer (excluding skin cancer)						
No				1.00		
Yes				1.61	1.05-2.48	0.03
						
Current smoking						
No				1.00		
Yes				0.97	0.68-1.40	0.89
						
Hemoglobin, g/dL				0.98	0.87-1.11	0.79
						
Serum albumin, g/dL				0.33	0.19-0.56	0.0001
						
Painful knees, no.						
0				1.00		
1				1.31	0.80-2.14	0.28
2				1.26	0.80-1.97	0.32
						
BMI^†^, kg/m^2^						
< 18.5				2.24	0.97-5.16	0.06
18.5-24.9				1.00		
25-29.9				1.12	0.76-1.67	0.56
≥ 30				1.14	0.73-1.78	0.57
						
SMI^‡^, %				1.01	0.97-1.06	0.57

*Q1, 2, 3, 4 represent 1^st ^through 4^th ^quartiles, from best performance (Q1) to worst performance (Q4).

^†^BMI = body mass index.

^‡^SMI = skeletal muscle index.

### Association with other physical performance tests

Self-reported limitation in rising from a chair was associated not only with performance on the chair rise test, but also with performance on the 8-foot walk and with limitation in hip and knee flexion, when these physical performance tests were included in the model (**Table **5). Associations with self-reported limitations getting in or out of bed were similar. Poor performance on the 8-foot walk test as well as limitations in hip and knee flexion were significantly associated with self-reported difficulty walking between rooms. These findings indicate that performance tests were not uniquely specific in explaining variation in corresponding self-reported functional limitations.

**Table 5:** Association of related and unrelated physical performance tests with self-reported physical function in multivariate models*

*Self-Reported Physical Function*										
		**Model 1**			**Model 2**			**Model 3**		
				
		**Rise from Chair**			**Get in or out of Bed**			**Walk across Room**		
		**OR**^**†**^	**95% CI**	**P**	**OR**^**†**^	**95% CI**	**P**	**OR**^**†**^	**95% CI**	**P**
				
	Chair rise^‡^, sec									
Performance Measure	Q1 (2.0-11.1)	**1.00**			**1.00**			1.00		
	Q2 (11.2-13.6)	**1.55**	**1.09-2.21**	**0.01**	**1.13**	**0.74-1.72**	**0.56**	0.81	0.38-1.72	0.58
	Q3 (13.7-17.5)	**1.79**	**1.28-2.49**	**0.0007**	**1.63**	**1.08-2.46**	**0.02**	0.69	0.36-1.32	0.26
	Q4 (17.6-100)	**2.94**	**1.94-4.44**	**< 0.0001**	**2.45**	**1.57-3.82**	**< 0.0001**	1.37	0.69-2.70	0.37
										
	8-foot walk^‡^, m/sec									
	Q1 (≥ 0.91)	1.00			1.00			**1.00**		
	Q2 (0.71-0.90)	1.32	0.93-1.87	0.12	1.26	0.82-1.92	0.29	**1.37**	**0.64-2.90**	**0.42**
	Q3 (0.54-70)	1.61	1.14-2.28	0.007	1.53	1.00-2.35	0.05	**2.21**	**0.92-5.31**	**0.07**
	Q4 (≤ 0.53)	2.52	1.73-3.68	< 0.0001	2.58	1.52-4.38	0.0005	**7.84**	**3.73-16.49**	**< 0.0001**
										
	Lock & key^‡^, sec									
	Q1 (2.0-4.2)	1.00			1.00			1.00		
	Q2 (4.3-6.3)	0.65	0.50-0.84	0.0008	0.88	0.58-1.31	0.52	0.49	0.25-0.96	0.04
	Q3 (6.4-10.3)	1.04	0.79-1.38	0.77	1.18	0.80-1.72	0.41	1.14	0.61-2.13	0.68
	Q4 (10.4-60.0)	1.24	0.97-1.59	0.09	1.52	0.99-2.33	0.06	1.13	0.64-1.97	0.68
										
	Shoulder rotation^§^	1.24	0.88-1.73	0.22	1.29	0.87-1.91	0.20	1.06	0.64-1.74	0.82
										
	Hip/knee flexion^¶^	3.05	1.91-4.87	< 0.0001	2.27	1.48-3.49	0.0002	3.14	1.66-5.95	0.0004

*Values in bold represent physical performance test related to corresponding self-reported physical function.

^†^Odds ratio (OR) adjusted for age, gender, education, arthritis, stroke, diabetes mellitus, chronic bronchitis, emphysema, asthma, myocardial infarction, congestive heart failure, cancer (excluding skin cancer), smoking, hemoglobin, serum albumin, knee pain, body mass index, skeletal muscle index, and physical performance test.

^‡ ^Q1, 2, 3, 4 represent 1st through 4th quartiles, from best performance (Q1) to worst performance (Q4).

^§ ^Full shoulder rotation versus any limitation in shoulder rotation.

^¶ ^Full hip and knee flexion versus any limitation in hip and knee flexion.

### Complete case analysis

Results of complete case analysis were similar to those of the main analysis that used multiple imputation of missing values. In the complete case analyses, self-reported limitations rising from a chair were associated not only with worse performance on the repeated chair rise test, but also with older age, current cigarette smoking, arthritis, stroke, myocardial infarction, congestive heart failure, lower BMI, knee pain, and lower serum albumin level. Self-reported limitations getting in or out of bed were associated with worse performance on the chair rise test, arthritis, stroke, chronic bronchitis, congestive heart failure, and bilateral knee pain. Worse performance on the 8-foot walk test was associated with higher odds of self-reported limitations walking between rooms. Additional significant covariates included arthritis, stroke, chronic bronchitis, congestive heart failure, and knee pain. These results indicate that self-reports were influenced by personal and health characteristics and not exclusively by the measured difficulty in performing the task.

## Discussion

Our findings indicate that self-reported limitations in physical function were associated with measured performance on the task being assessed. Nonetheless, self-reported physical functioning was influenced also by personal and health characteristics and not solely by the measured difficulty in performing the task. These findings indicate that self-report captures information above and beyond performance on the specific task itself.

Functional limitations were strongly associated with physical performance tests, particularly for participants in the worst-performing quartiles. Despite the importance of physical performance tests, other factors were independently associated with self-reported physical functioning. Advanced age showed strong graded associations with limitations in each of the three functions. Participants with comorbid conditions were more likely to report worse limitations, consistent with prior reports [36,37]. For all three functions, serum albumin level was an important indicator of worse self-reported functioning, beyond the information on disease burden provided by the presence of comorbidities. Low serum albumin level has been associated with an increased odds of functional limitations in earlier studies [28,29]. Underweight participants had increased risks of self-reported limitations than their normal weight counterparts, consistent with previous reports that low BMI is associated with functional limitations [31,38]. Pain in both knees was significantly associated with an increased odds of limitations rising from a chair and getting in or out of bed. These findings suggest that self-reports of functional limitations represent global perceptions of frailty, rather than solely an appraisal of limitations on the task being asked.

Despite extensive literature on this topic, the nature of the association between physical performance tests and self-reported limitations has remained incompletely characterized. Most prior studies compared a group of physical performance tests (typically a performance battery) with multi-item self-report functions [14,15,18-20,39]. For example, Reuben and colleagues found weak associations between physical function questionnaires and a battery of physical performance tests in 83 older adults [14]. Myers and colleagues found good correspondence (defined as > 80% agreement) between a set of 14 physical performance tests and a set of corresponding self-reported limitations in only one-third of participants [13]. We similarly found that physical performance tests did not correspond exclusively to self-reported limitations. Kempen et al studied the relationship of sociodemographic characteristics, performance tests, personality measures, and cognitive and affective functioning and self-reported limitations in 753 older adults [17]. They found that associations between physical performance tests and corresponding self-reported limitations were weak, and that some of the discrepancy was explained by depressive symptoms and self-efficacy. These results support our findings in suggesting that factors other than performance can impact self-report. However, while they accounted for cognitive and affective symptoms, we found that less well-recognized physical factors, such as serum albumin level, pain, and BMI were associated with a higher odds of self-reported limitations.

Physical performance tests that were not specifically paired to the physical functions studied were also significantly associated with self-reported limitations. For example, poor performance on the 8-foot walk test, a test of gait, was associated with worse self-reported functioning in rising from a chair and getting in or out of bed, which are measures of changes in body position. These associations demonstrate performance tests were not exclusive correlates of their specifically paired self-reported limitations, and that tests of other lower extremity functions (but not of upper extremity functions) also influence self-reports.

In contrast to many previous studies, we used highly specific pairings to provide a more valid test of the relationship between physical performance tests and self-reported physical function, thereby minimizing problems of compensability and the risk of confounding across different domains of physical functioning. We also tested a broad set of personal and health characteristics as correlates. Moreover, the population-based national sample increases the generalizability of our results.

Our study also has some limitations. Because we wanted to examine self-reported functions for which there were corresponding physical performance tests, we were able to examine only two physical performance tests. Although we do not know if our results apply to other physical performance tests, the consistency of results suggests that the findings may be relevant for other physical functions. We did not have data on depressive symptoms, personality measures, fatigue, and cognitive functioning, each of which can affect physical functioning [15]. However, our objective was not to identify all factors that may impact self-reported physical functioning, but rather to test the relationship between physical performance and patient factors and self-reported functioning. Although physical performance tests were related to self-reported limitations, measurement error and the effort-dependence of performance measures may have led to an underestimate of these associations. Our sample included persons aged ≥ 60 years, and we do not know if the associations are generalizable to younger individuals.

## Conclusions

Our results support the validity of self-reported physical function by finding associations between self-reports and measured performance on similar tasks. More important, however, were our findings that these associations were neither specific nor exclusive. Personal and health characteristics of respondents also influenced self-reported physical function. Our findings caution against a narrow or strict interpretation of self-reported limitations in individual tasks. Self-reported limitations represent a gestalt rather than an appraisal isolated from its context. Self-reports capture information beyond task difficulty.

## Competing interests

The authors declare that they have no competing interests.

## Authors' contributions

GL and MW conceived of the study, designed the study, performed the statistical analysis, interpreted the results, and drafted the manuscript. All authors read and approved the final manuscript.
